# Isotretinoin, Vitamin A Supplements, and Unintended Pregnancies in Post Roe v. Wade America

**DOI:** 10.7759/cureus.31442

**Published:** 2022-11-13

**Authors:** Praneet S Paidisetty, Leonard K Wang, Dina H Zamil, Shangyi Fu, Zeena Y Nawas

**Affiliations:** 1 Dermatology, McGovern Medical School, The University of Texas Health Science Center, Houston, USA; 2 Dermatology, John Sealy School of Medicine, The University of Texas Medical Branch, Galveston, USA; 3 Dermatology, Baylor College of Medicine, Houston, USA

**Keywords:** abortion, teratogenic medications, ethics, acne, reproductive autonomy, vitamin a supplements, isotretinoin

## Abstract

Isotretinoin is a potent vitamin A derivative that is used to treat acne. However, despite its utility in dermatologic care, it is also highly teratogenic and can cause severe life-threatening fetal abnormalities in the first trimester of pregnancy. As a result, existing regulations are stringent in order to prevent accidental pregnancies in women taking isotretinoin. In the unlikely case of an unintended pregnancy, while taking isotretinoin, a woman could terminate her pregnancy with an abortion. However, with the recent overturning of *Roe v. Wade*, we explore the consequences of this landmark United States Supreme Court ruling with special attention to those taking isotretinoin and over-the-counter (OTC) vitamin A supplements.

## Editorial

New restrictive abortion laws passed in response to the overturning of *Roe v. Wade* may increase the number of births carried to term. A greater number of full-term births likely will lead to an absolute increase in congenital complications, as around two to four percent of all neonates have inborn defects, many of which are preventable [1]. Modifiable risks for such defects include substances, supplements, and medications, the use of which is especially prevalent in at-risk populations with low socioeconomic status [1]. 

Isotretinoin is one such medication (Accutane, Roche Pharmaceutical, Basel, Switzerland). In 2006, the United States Food and Drug Administration (FDA) developed the iPledge Risk Evaluation and Mitigation Strategy (REMS), a stringent electronic registry to prevent birth defects caused by the acne drug, isotretinoin. For many years, it was unknown whether the FDA’s efforts to counteract the teratogenicity of isotretinoin were effective. However, Tkachenko et al. reported that there were 6,740 pregnancies among women on isotretinoin from 1997 to 2017, with a peak incidence of 0.65% in 2006 [2]. Though the number of pregnancies among women taking isotretinoin has decreased since its peak, pregnancies persist in the iPledge REMS era.

Such pregnancies lead to life-threatening birth defects, such as central nervous system abnormalities, heart and craniofacial defects, and endocrine irregularities [1]. If a woman becomes pregnant while taking isotretinoin, she generally would have the option of avoiding deleterious fetal defects by undergoing an abortion. But, the recent overturning of *Roe v. Wade* by the United States Supreme Court has proved to have pervasive effects on women’s management of their fertility and reproductive status. In awaiting this Supreme Court decision, several states had “trigger laws” in place to immediately outlaw abortions with far fewer exceptions than current laws and other states had similar pending legislation. As such, there is no current federal protection afforded to unintended pregnancies in women taking isotretinoin, although state-level legislation may differ. 

Isotretinoin is not the only product used for acne that poses a risk to pregnant women and their fetuses. Over-the-counter (OTC) supplements marketed for acne treatment can contain high doses of vitamin A. The FDA does not currently regulate these easily-accessible vitamin A supplements despite their teratogenic potential and side effects, and such products often do not display pregnancy warnings [3]. Both isotretinoin (regulated by the FDA) and vitamin A acne supplements (not regulated by the FDA) pose significant safety concerns to female patients of reproductive age, especially in light of the Supreme Court’s decision to overturn *Roe v. Wade*.

Patients with acne who do not have insurance or financial means to consult a dermatologist may use OTC supplements that have unregulated claims and contents. Supplement ingredients might include retinoic acid, folic acid antagonists, heavy metals, or other pollutants or contaminants, which pose an increased risk of birth defects [1]. In some cases, patients may attempt to reproduce the effects of Accutane by consuming high doses of OTC vitamin A supplements, risking hepatotoxicity and congenital birth defects [1]. The burden of disease on parents and their newborns can financially stress a family that already has limited resources, precipitating lifelong financial and medical disability [4].

Furthermore, vitamin A supplements, which may pose a similar risk of birth defects as isotretinoin, often display Supplement Facts labels that can be difficult to interpret [3]. For consumers to accurately determine if a given supplement contains a teratogenic level of vitamin A, they need to know the teratogenic dose threshold, which is not straightforward for the average person to find, understand, or calculate. For example, buried within the medical literature, the teratogenic threshold for vitamin A dose is provided in International Units (IUs) [3,5]. Because the FDA currently requires manufacturers to provide vitamin A doses in units of Retinol Activity Equivalents (RAEs), consumers need to convert from IUs to RAEs. The instructions for this conversion can be found online but are best detailed in articles intended for healthcare professionals, not the general public [3]. Additionally, patients in the United States also may not be aware a teratogenic threshold exists, let alone that it can be found in clinically-oriented resources. Finally, not all supplements will contain all the information necessary to perform the aforementioned calculations. In fact, previous literature has noted that teratogenic risk could not be determined from the labels of vitamin A-containing supplements sold online [3]. Performing such calculations may be difficult for individuals with poor health literacy and limited socioeconomic resources. This may result in the use of such supplements during pregnancy, leading to an increased risk for negative effects on fetal development. Thus, in the context of vitamin A derivatives, abortion laws are poised to perhaps disproportionately affect individuals of low socioeconomic status in America because they may not be aware of the teratogenicity of OTC acne supplements or have the knowledge or resources to consult a health professional prior to consumption.

The overturning of *Roe v. Wade* has and will continue to create significant shifts in how women manage their fertility. Unfortunately, this landmark ruling has unjustly created inequities in the management of incidental pregnancies for those who take isotretinoin and vitamin A supplements. Potential steps forward include community outreach in vulnerable areas and encouraging organized public health groups to lobby pertinent political entities to bring attention to this problem. Finally, we strongly advocate for increased discussion and research to fight for the reproductive rights of women consuming potentially teratogenic medicines, nevertheless for all women.

## References

[1] Harris BS, Bishop KC, Kemeny HR, Walker JS, Rhee E, Kuller JA (2017). Risk factors for birth defects. Obstet Gynecol Surv.

[2] Tkachenko E, Singer S, Sharma P, Barbieri J, Mostaghimi A (2019). US food and drug administration reports of pregnancy and pregnancy-related adverse events associated with isotretinoin. JAMA Dermatol.

[3] Zamil DH, Burns EK, Perez-Sanchez A, Katta R (2022). Do you know how to assess risks posed by over-the-counter vitamin A supplements?. AMA J Ethics.

[4] Lemacks J, Fowles K, Mateus A, Thomas K (2013). Insights from parents about caring for a child with birth defects. Int J Environ Res Public Health.

[5] Rothman KJ, Moore LL, Singer MR, Nguyen US, Mannino S, Milunsky A (1995). Teratogenicity of high vitamin A intake. N Engl J Med.

